# Neuroscience needs creativity: the implications of reliable instruments that fail to measure a loosely defined latent variable

**DOI:** 10.3389/fnhum.2014.00869

**Published:** 2014-10-31

**Authors:** Chiara S. Haller

**Affiliations:** ^1^Department of Psychology, Harvard UniversityCambridge, MA, USA; ^2^Harvard Medical SchoolBoston, MA, USA; ^3^Division of Public Psychiatry, Massachusetts Mental Health CenterBoston, MA, USA; ^4^Department of Psychiatry, Beth Israel Deaconess Medical CenterBoston, MA, USA

**Keywords:** creativity, neuroscience, validity and reliability, multidisciplinary approach, the Holy Grail

## Why investigate creativity?

To make a better world, correcting mistakes is not enough. We need positive ambition that motivates us to go on. Creativity (i.e., the process of generating something new and useful), which offers the most exciting models of life, is such a source of motivation. Without thinking about fame or prestige, creativity makes people want to change something in the world. Through research on pathological cases psychologists have discovered a great deal about the thinking and feeling of healthy people. But not much has been learnt about those at the other end of the scale, namely those significantly above average in a positive sense. If people want to find out what is missing in their life, they should consider finding out more about those people who possess what they are missing, that is who are creative and happy.

## How to investigate creativity

Psychologists began to investigate creativity in the 1950s, and had many problems finding a widely accepted definition of it. They began to construct paper-and-pencil tests to measure the creative potential of people. Some of the characteristics more commonly found were tolerance of ambiguity, openness to experience, flow, autonomy, verbal fluency, metaphoric thinking, persistence and intrinsic motivation, as well as complexity, which is defined as a confluence of conflicting attributes in the same person (e.g., extraversion and introversion) and the flexibility to switch back and forth among these attributes, as the situation requires (Haller and Courvoisier, 2010).

Most of the studies found that the most important characteristic of creative individuals is “an almost esthetic ability to recognize a good problem in their domain” (Sawyer, 2006, p. 47). What these results suggest is that the creative personality is somehow related to a specific domain. Individuals possess different possible features, but the confluence of certain characteristics may be more valuable in one domain than in another (e.g., a musician may need to be more extraverted than a visual artist). Conventions in different domains (e.g., art vs. science) most obviously are not the same, and a person might choose a domain that fits his or her predispositions.

When Amabile (Amabile, 1982; Hill and Amabile, 1993) analyzed the first wave of tests, which measured the originality of individuals (e.g., Torrance Test of Creative Thinking; Torrance, 1998), she also noticed that there was always a subjective, implicit assessment built into those tests. Originality was not captured objectively, but through the validation of raters, who had their own subjective criteria. Amabile concluded that: “A product is creative when experts in the domain agree it is creative” (p. 1001).

This gave rise to one of the most frequently reported techniques for assessing the creativity of a product in a specific domain, that is, independent expert evaluation of a work as more or less creative. However, these efforts seemed to suffer from subjectivity. How can we know who is appropriate as an expert? Some researchers (Haller et al., 2010, 2011) found that the rating of the products changed according to the creator's characteristics (e.g., intelligence), and the background of the rater (e.g., expert vs. novice raters).

Cognitive psychologists were concerned with creative mental processes. They wanted to explain the phenomenon of creativity by pointing out that it develops from normal, daily mental processes, from cognitive abilities everyone possesses. The result is widespread knowledge about, and acceptance of the 5 stages of the creative process: (1) preparation, (2) incubation- or maturation, (3) illumination- or enlightenment, (4) evaluation, and (5) elaboration (Cropley and Cropley, 2010). These stages can differ in depth and breadth among subjects, cultures, and domains, and they can also repeat and overlap with variable intensity (Shaw, 1989). This process seems to underlie creation independently of the domain a person or group has to adjust to.

The investigation of brain function across time may call for scientific tools such as the electroencephalogram (EEG), which is a non-invasive measure of electrical brain activity. Electric field potentials are recorded that represent changes of potential differences between different areas that arise from either excitatory and/or inhibitory postsynaptic potentials. Whereas the P300 (300 ms after stimulus) measures cognitive processing speed and attentional resource allocation, N200 (200 ms after stimulus) measures cognitive response inhibition (Patel and Azzam, 2005). To measure P300, and/or N200, specific tasks have to be created, which the participant has to solve. A reaction to very specific auditory or visual stimuli however, has so far been an unsatisfactory method of investigation, and has mostly been used for insight studies (i.e., measuring the third stage of the creative process). After all, we cannot just have someone walk around with an EEG cap on, and measure the stages of the creative process in real time. Since short-time brain reactions are not suitable to detect changes in cortical activity, which presumably (as in the case of creativity) occur in long time windows, EEG may need to be complemented by other scientific methods.

A far more promising approach that involves EEG experiments is the comparison of artists and non-artists on a task closer to real-life (e.g., composing music, visualizing an abstract concept). The question arises however, whether these experiments simply prove domain specificity or actually capture creativity *per se*. It does not seem too unexpected that a musician shows different brain activity patterns from those of a non-musician when thinking about music. The question remains whether a scientist shows the same brain activity when thinking about science as the musician does when thinking about music.

Neuroimaging studies measuring functional aspects of brain activity—fMRI, PET, or NIRS—rely heavily on tests that measure remote associations or divergent thinking (i.e., variant thinking; e.g., TTCT). The origin of the former lies in the development of the Remote Association Test (RAT) on the basis of the idea that individuals have an *associative hierarchy*, which leads to more or less creative solutions (i.e., flatter associative hierarchies = more creative solutions) (Mednick, 1962). However, the resulting test asks for a single correct association implying cognitive processes similar to those from a wide range of other analytic and *convergent* thinking tests (Lee et al., 2014). Creativity may not be comparable to a stage of problem-solving, since individuals may work in domains where problems have not yet been specified. Thus, their success is dependent on the formulation of a new problem (Csikszentmihalyi, 1996; Runco, 2014).

Other association tests do not ask for a single solution, but for original associations with a specific word or letter. While verbal and/or semantic fluency measures do not ask for a certain solution, most of them are timed (Benedek and Neubauer, 2013). Thus, small changes—such as the time for completing the test—lead to higher or lower “creativity” scores within the same person (Barron and Harrington, 1981). While processing speed is an important component of *intelligence* it may be less important in the construct *creativity*. In fact, it may be one possible explanation of the U-shaped relationship between intelligence and creativity (i.e., threshold hypothesis). In other words while processing speed may show a linear relationship with intelligence, it may show only a partial correlation with creativity.

For any such task we think of working memory and executive attention, thus some kind of activation (or more probably *de* activation) of prefrontal regions (Chávez-Eakle et al., 2007; Fink et al., 2010). The question remains which prefrontal cortices are involved. Some studies point to specific areas (e.g., supplementary motor area, frontal gyrus [BA9], right premotor region [BA6], right ventromedial prefrontal cortex [BA11], frontal pole [BA10], left anterior cingulate cortex [ACC], left dorsolateral prefrontal area [BA46]), while others present diffuse prefrontal activation patterns. This inconsistency is to be expected, since even though the divergent thinking and association tasks used are made as simple as possible, they still engage more than one process. Thus, apart from the fact that neither divergent thinking nor associative thinking or convergent thinking by itself is synonymous with creativity, the process of creativity may change slightly according to the domain being investigated and the task at hand.

Similar to EEG, functional resonance imaging naturally restricts the possibilities of testing a real-life scenario. We are currently unable to create tasks that call for the complexity and thus time a reality-close creative endeavor would demand. Even an imagined real-life process (i.e., composition) will cause its problems. Similarly to the situation with EEG, we either look at individual differences, and thus prove or disprove domain specificity, or we target one specific brain area, using one specific task (e.g., verbal fluency as a proxy for divergent thinking, which is supposed to be a proxy for creativity), thus, proving nothing other than that brain area differs functionally among people on a specific task such as verbal fluency. Thus, brain imaging may very reliably measure the wrong thing.

The finding that general functions (e.g., WM) have been found to be involved in creativity tasks; and that the activation of brain areas that support such general function (but not creativity in particular) have been found to be activated in creativity tasks; underlines the problem of operationalization. It seems that creativity researchers agree more or less on the theoretical framework of creativity, although they fail when trying to develop a task that captures the holy in its Holiness. One possible solution may be to omit the term *creativity* altogether. Instead of wanting to find a single measure that captures creativity, researchers may try to discuss possible relationships between different functions (e.g., verbal fluency, brain functions and the amount to which these functions are exhibited) and their respective relations that may lead a person to create something new in a specific area of expertise.

## The Holy Grail of creativity

Research on creativity has blossomed over the past 60 years, and investigations in different areas of psychology, as well as history, anthropology, sociology, and research in neuroscience have made remarkable progress over the past few decades. The question remains, however, which brain *circuits* do we look at in order to investigate the creative process. We know that the seat of cognitive flexibility lies in the frontal lobe, and both hypofunction and gray matter loss in fronto-temporal and parietal regions are linked to neurocognitive impairments. It seems reasonable to suggest that greater functioning in frontoparietal and temporal regions, including the cingulate cortex (left anterior, BA 32), hippocampus and amygdala, dorsolateral regions (Brodmann areas [BA] 8, 9, and 47), lingual gyrus, temporal pole and orbitofrontal regions are related to creativity. The question remains, *how* these structures are related, what role personality plays, what the difference across people and domains is, and how they differ across cultures and historically. We recently commenced large-scale data collection online (http://creativity.wjh.harvard.edu) in an attempt to address this issue.

Rather than asking *if* a neuroscience of creativity is needed, we need to ask *how* it can be achieved and integrated into a combined approach. Rather than looking for one special ingredient, the magic pill we can take that makes us creative; we may want to ask what confluence of factors leads us to become creative in a specific domain. There is no magic pill, no Holy Grail, no one brain area, no one personality, or one way to solve a problem. Creativity is a lot of work, emotionally involving, requires motivation, commitment, persistence, curiosity, love of learning, authenticity, and honesty, working memory, executive, prefrontal and limbic system function. The confluence of some of these different ingredients may change according to the domain involved (e.g., a contemporary dancer may use more of his or her limbic system, whereas a neuroscientist may need more of his or her executive function), whereas some factors may be more universal (e.g., complexity, cognitive flexibility). It seems to be quite clear that neither research area by itself will solve the puzzle, nor will any single brain function explain the results. We need to stop looking for the single Holy Grail and to start looking for the multiple ingredients that creativity is comprised of. Creativity—like water—changes its form as needed, and continuously, patiently, and passionately finds its way through the rocks.

### Conflict of interest statement

The author declares that the research was conducted in the absence of any commercial or financial relationships that could be construed as a potential conflict of interest.
